# Distinctive outcome in patients with non-uterine and uterine leiomyosarcoma

**DOI:** 10.1186/1471-2407-14-981

**Published:** 2014-12-18

**Authors:** Wolfgang Lamm, Camilla Natter, Sophie Schur, Wolfgang J Köstler, Alexander Reinthaller, Michael Krainer, Christoph Grimm, Reinhard Horvath, Gabriele Amann, Philipp Funovics, Thomas Brodowicz, Stephan Polterauer

**Affiliations:** Clinical Division of Oncology, Department of Medicine I, Medical University of Vienna, 18-20 Waehringer, Guertel, 1090 Vienna Austria; Department of Gynecology and Gynecologic Oncology, Medical University of Vienna, 18-20 Waehringer, Guertel, 1090 Vienna Austria; Department of Pathology, Medical University of Vienna, 18-20 Waehringer, Guertel, 1090 Vienna Austria; Comprehensive Cancer Center, Medical University of Vienna, 18-20 Waehringer, Guertel, 1090 Vienna Austria; Department of Orthopedics, Medical University of Vienna, 18-20 Waehringer, Guertel, 1090 Vienna Austria; Sarcoma Platform, Vienna, Austria; Medical University of Vienna, Gynecologic Cancer Unit - Comprehensive Cancer Center Vienna, 18-20 Waehringer, Guertel, 1090 Vienna Austria

**Keywords:** Outcome, NULMS, ULMS, Prognostic factors

## Abstract

**Background:**

Leiomyosarcomas represent the largest subtype of soft tissue sarcomas. Two subgroups can be distinguished, non-uterine (NULMS) and uterine leiomyosarcomas (ULMS). The aim of this retrospective study was to evaluate differences in clinical features and outcome between these two subgroups.

**Methods:**

Outcome and clinical-pathological parameters between 50 patients with NULMS and 45 patients with ULMS were assessed, and compared between both groups. Univariate and multivariable survival analyses were performed.

**Results:**

Patients with ULMS presented with larger tumors when compared to patients with NULMS (p < 0.001). More patients with ULMS initially presented with metastatic disease (67% vs. 36%, p = 0.007). Most common metastatic site was lung for both subtypes (28% and 38%). Five-year overall survival (OS) rates of 82.6% and 41.2% and median OS times of 92.6 (range: 79.7-105.4) and 50.4 (range: 34.8-66.0) months were observed in patients with NULMS and ULMS, respectively (p = 0.006). In multivariate analysis, initial metastatic disease remained an independent prognostic factor in terms of OS (p < 0.0001).

**Conclusion:**

At time of diagnosis ULMS were larger and more often metastasized. Therefore patients with ULMS showed unfavorable outcome when compared to NULMS. Later diagnosis might be caused by differences in symptoms and clinical presentation or a more aggressive biological tumor behavior.

## Background

Leiomyosarcomas (LMS) are a rare large subgroup of all soft tissue sarcomas (STS) that account for approximately 24% of all STS. LMS is a mesenchymal tumor of smooth muscle origin found in the uterus and in soft tissue [1]. LMS can occur anywhere in the body, but the most frequently affected organs are the retroperitoneal space, the extremities (NULMS) and the uterus (ULMS). ULMS is the most common uterine sarcoma and accounts for 40% of all uterine sarcomas [2]. In contrast to epithelial endometrial cancer, overall prognosis is still poor [3].

Management of initially localized NULMS consists of complete surgical resection and radiation therapy with overall survival of approximately 8-13 months [4]. Different chemotherapy regimens in the front line as well in the palliative setting showed promising efficacy in first line as well in advanced disease [5–7]. Total hysterectomy and bilateral salpingo-oophorectomy is the initial treatment for women with ULMS. The role of pelvic lymphadenectomy is unclear and is recommended when intraoperatively palpable lymph nodes are present or in women with extrauterine disease. For women with disease that has spread beyond the uterus but is confined to the peritoneal cavity surgical cytoreduction is recommended. In patients with metastatic disease that cannot be completely surgically resected administration of chemotherapy is favored over adjuvant radiation therapy RT. Radiation therapy does not show any benefit in early stage disease [8, 9]. Chemotherapy in first line as well in advanced disease has shown promising results [10–12].

So far the outcome between patients with NULMS and ULMS seems to be different, but high quality data is limited. In the largest study so far Oosten et al. reported outcomes of first line chemotherapy in patients with advanced or metastatic NULMS and ULMS [13]. Interestingly, no significant differences in outcomes for uterine and non-uterine LMS were reported in their report.

Thus, the aim of this retrospective study was to evaluate the different outcomes between NULMS and ULMS compromising all stages.

## Methods

### Patients

In total, 50 (53%) patients with NULMS (superficial LMS were excluded) and 45 (47%) patients with ULMS were included in this retrospective multicenter study (Department of Gynecology and Gynecologic Oncology, Medical University of Vienna, Vienna, Austria: n = 16; Department of Oncology, Medical University of Vienna, Vienna, Austria: n = 79) between 1998 and 2013. Data were collected by chart review. For NULMS, tumor grading was based on the French Federation of Cancer Centers Sarcoma Group (FNCLCC) grading system, a three-grade classification published by Coindre [14]. The International Federation of Gynecology and Obstetrics (FIGO) developed a new classification system especially for ULMS to include different variables like tumor size, extra uterine spread and invasion of abdominal tissues [13]. Primary tumor assessment was done using magnetic resonance imagining (MRI) and/or computed tomography (CT) and clinical examination. Screening for distant metastasis was done using CT scans of the chest and abdomen. Follow up care with CT scans of the chest and abdomen and MRI of the primary located tumor was done every three to four months for the first three years, every six months up to the year five and annually thereafter. All patients consented to treatment according to institutional guidelines, and all patients had consented to anonymized assessments and analysis of data and outcome of therapy.

### Treatment

Treatment consisted of surgical resection of primary tumor (NULMS: surgical resection of primary disease, in case of incomplete resection margins, re-resection within 1 month was performed; ULMS: including hysterectomy, bilateral salpingo-oophorectomy, pelvic and/or paraaortic lymphadenectomy in presence of intraoperatively palpable lymph nodes and surgical cytoreduction in women with extrauterine disease). If clinically indicated, radiation therapy, adjuvant as well as palliative chemotherapy was recommended. Radiation therapy was performed as 3D CT based conformal therapy. Adjuvant as well as palliative chemotherapy regimens was used according to local practice.

All patients consented to treatment according to institutional guidelines, and all patients had consented to anonymized assessments and analysis of data regarding outcome of therapy. The local ethical committee of the Medical University of Vienna approved all analyses.

### Statistical analysis

For statistical analysis, we used Statistical Package for the Social Sciences (SPSS) software (SPSS 21.0; SPSS Inc., Chicago, Illinois). Clinico-pathological parameters were compared between NULMS and ULMS using Students’ t-test, chi-square tests, and One-way ANOVA analyses where appropriate. Overall survival (OS) was calculated from the data of primary diagnosis until death or last follow-up. OS was presented by using Kaplan-Meier curves and differences between NULMS and ULMS patients were compared by log-rank test. Univariate survival analyses were performed, compromising patients’ mean age (≤56.6 vs. >56.6 years), gender (male vs. female), histologic grade (G1 vs. G2 + 3), Tumor size (≤5 cm vs. 5-10 cm vs. >10.0 cm), P-values <0.05 were considered statistically significant. All parameters showing a significant (p < 0.05) prognostic effect were included into a multivariable Cox-regression analysis. An event was defined as death. Radiologic response to adjuvant chemotherapy was assessed according to RECIST criteria: complete response (CR) – no measurable disease; partial response (PR) - greater than 50% response; stable disease (SD) – less than 25% response or no response and progressive disease (PD) [15]. Overall response rate (ORR) was defined as the proportion of patients whose best overall response was either CR or PR.

## Results

### Baseline characteristics for all patients

Baseline characteristics of patients with NULMS and ULMS are outlined in Table 1. Fifty patients (female: n = 22; male: n = 28) presented with NULMS and 45 female patients presented with ULMS. For patients with NULMS, most common tumor site was the extremity (60%) followed by the abdomen (32%). Fifteen patients with ULMS (33%) presented with FIGO stage I, 3 patients (7%) with FIGO stage III and 27 patients (60%) with FIGO stage IV. Twenty-three patients (46%) had a tumor size ≤5 cm and 37 patients (74%) had a deep tumor location. Nineteen patients (42%) had a tumor size greater than 10.0 cm. Thus, at the time of diagnosis patients with ULMS had greater tumor size when compared to NULMS (>10.0 cm: 42% ULMS vs. 10% NULMS, p < 0.001). More patients with ULMS presented with initial metastatic disease (67% vs. 36%, p = 0.007). Most common metastatic sites were lung (28% vs. 38%), followed by liver (14%) in NULMS and pelvic/paraaortic lymph nodes (18%) in ULMS. Patients with NULMS and ULMS with initially metastatic disease receiving chemotherapy showed overall response rates of 33% and 18% and clinical benefit rates of 55% and 33%, respectively.

**Table 1:** **Baseline characteristics**

	NULMS	ULMS	p value
	n (%) or median (range)	n (%) or median (range)	
**N**	50 (100%)	45 (100%)	
**Age at first diagnosis**	61 (31-88)	54 (32-69)	0.002
**Gender**			<0.001
- Male	28 (56%)		
- Female	22 (44%)	45 (100%)	
**Tumor stage**			
- FIGO I		15 (33%)	
- FIGO III		3 (7%)	
- FIGO IV		27 (60%)	
**Histological grade**	**n = 47**	**n = 34**	0.027
- G 1	1 (2%)	3 (9%)	
- G 2 + 3	46 (98%)	31 (91%)	
**Tumor site**	**n = 50**		
- Extremities	30 (60%)		
- Abdominal	16 (32%)		
- Head/neck	2 (4%)		
- Thorax	2 (4%)		
**Tumor size**	**n = 50**	**n = 45**	<0.001
cm	11.1 (3-40)	6.7 (3-14)	
- ≤5 cm	23 (46%)	6 (13%)	
- 5-10 cm	22 (44%)	14 (32%)	
- > 10.0 cm	5 (10%)	19 (42%)	
- Not evaluable		6 (13%)	
**Tumor location**	**n = 50**		
- Deep/superficial	37 (74%)/13 (26%)		
**Initial metastatic disease**			0.007
- Yes	18 (36%)	30 (67%)	
**Metastatic sites**	**n = 18**	**n = 30**	
- Lung	14 (28%)	17 (38%)	
- Liver	7 (14%)	5 (11%)	
- Bone	4 (8%)	4 (9%)	
- Lymph nodes	3 (6%)	8 (18%)	
- Other	5 (10%)	15 (33%)	
**Surgical resection**			0.2
- Yes	47 (94%)	45 (100%)	
**Resection margins**	**n = 47**	**n = 45**	0.048
- Wide	45 (96%)	37 (82%)	
- Marginal	2 (4%)	8 (18%)	
**Re-resection**			
- Yes	16 (34%)		
**Pulmonary metastaseectomy**			0.6
- Yes	6 (12%)	8 (18%)	
**Radiationtherapy**			0.04
- Yes	29 (58%)	16 (36%)	
**Adjuvant Chemotherapy for initially localized disease**			
- Yes	13 (41%)	8 (53%)	
**Chemotherapy Regimen for initially localized disease**			
- Epirubicine/ifosfamide	6 (46%)	3 (37%)	
- Doxorubicine	5 (39%)	1 (13%)	
- IFADIC	2 (15%)	2 (25%)	
- Gemcitabine/docetaxel	0	2 (25%)	
**Relapse**			
- No	12 (92%)	6 (75%)	
- Yes	1 (8%)	2 (25%)	
**First line chemotherapy for initially metastastic disease**			
- Yes	18 (36%)	27 (60%)	
**First line chemotherapy regimen for initially metastastic disease**	**n = 18**	**n = 27**	
- Doxorubicine	9 (50%)	7 (27%)	
- Epirubicine/ifosfamide	6 (33%)	2 (7%)	
- IFADIC	0	3 (11%)	
- Other	3 (17%)	2 (7%)	
- Gemcitabine/docetaxel		13 (48%)	
**Response to first line Chemotherapy for initially metastastic disease**	**n = 18**	**n = 27**	
- Complete remission	4 (22%)	2 (7%)	
- Partial remission	2 (11%)	3 (11%)	
- Stable disease	4 (22%)	4 (15%)	
- Progressive disease	7 (39%)	18 (67%)	
- Not evaluable	1 (6%)		
**Follow-up time (months)**	50 (1.2-153.7)	33 (1.5-269.1)	
**Disease status**			
**Alive/dead**	38 (76%)/12 (24%)	20 (44%)/25 (56%)	

*Patients had initially localized disease; NULMS = non-uterine leiomyosarcoma; ULMS = uterine leiomyosarcoma.

IFADIC = ifosfamide-doxorubicin-dacarbazine.

### Patients’ characteristics with NULMS

Baseline characteristics are outlined in Table 1. Thirty-two patients (64%) presented with initial localized disease and 13 patients (41%) out of these 32 patients received chemotherapy. Eighteen patients (36%) presented with initial metastatic disease. Most common metastatic sites were lung (28%), followed by liver (14%). Surgery resection of primary disease was performed in 47 patients (94%). Repeat resection, due to marginal resection margins within 1 month after primary tumor resection was necessary in 16 patients (34%). Pulmonary metastasectomy was performed in 6 patients (12%). Palliative radiation therapy and chemotherapy for initial metastatic disease was performed in 29 (58%) and 18 (36%) patients, respectively. Most common first line palliative chemotherapy regimen for initial metastatic disease in these 18 patients was doxorubicin (50%). Seven patients (39%) relapsed after first therapy necessitating further palliative therapy. Twelve out of 50 patients (24%) died. Median follow up of all patients was 50 months (range: 1.2-153.7).

### Patients’ characteristics with ULMS

Baseline characteristics are described in Table 1. Fifteen patients (33%) presented with initial localized disease and 30 patients (67%) presented with initial metastatic disease. Most metastatic sites were pulmonary (38%) followed by lymph nodes (18%) and liver (11%). All patients underwent surgical resection of primary disease with wide resection margins (surgical plane is in normal tissue) in 37 patients (82%). Pulmonary metastasectomy was performed in 8 patients (18%). Sixteen patients (36%) underwent radiation therapy. Palliative chemotherapy for initial metastatic disease was performed in 27 patients (60%). Most common palliative first line chemotherapy regimen of these 27 patients was gemcitabine/docetaxel (48%). The ORR after first line palliative chemotherapy was 18%. Twenty-five out of 45 patients died with a median follow up time of 33 months (range: 1.5-269.1).

### Survival

**Figure 1:**
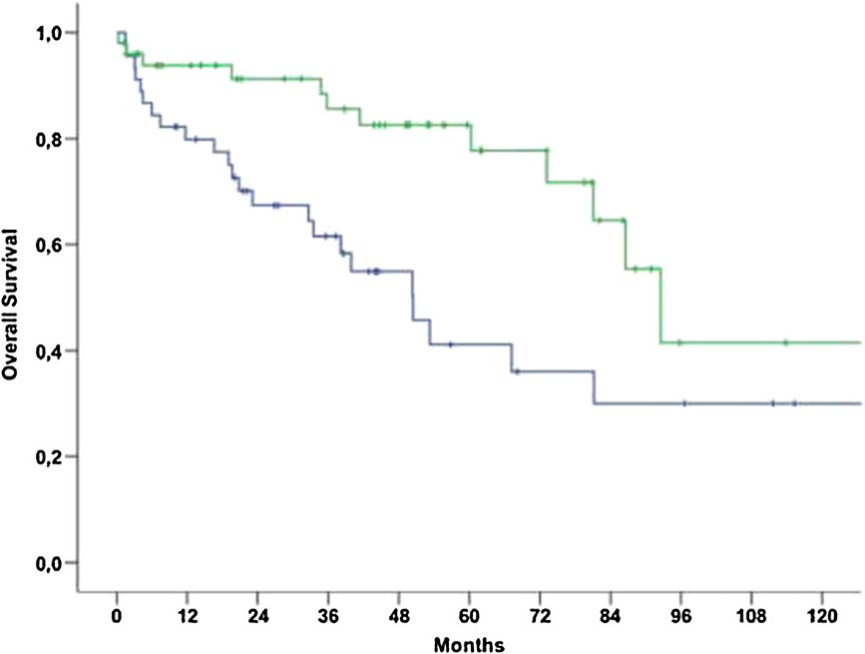
**Overall survival for patients with NULMS (green curve) vs. ULMS (blue curve) (p = 0.006); 5 yr OSR 82.6% (NULMS) vs. 41.2% (ULMS).**

**Figure 2:**
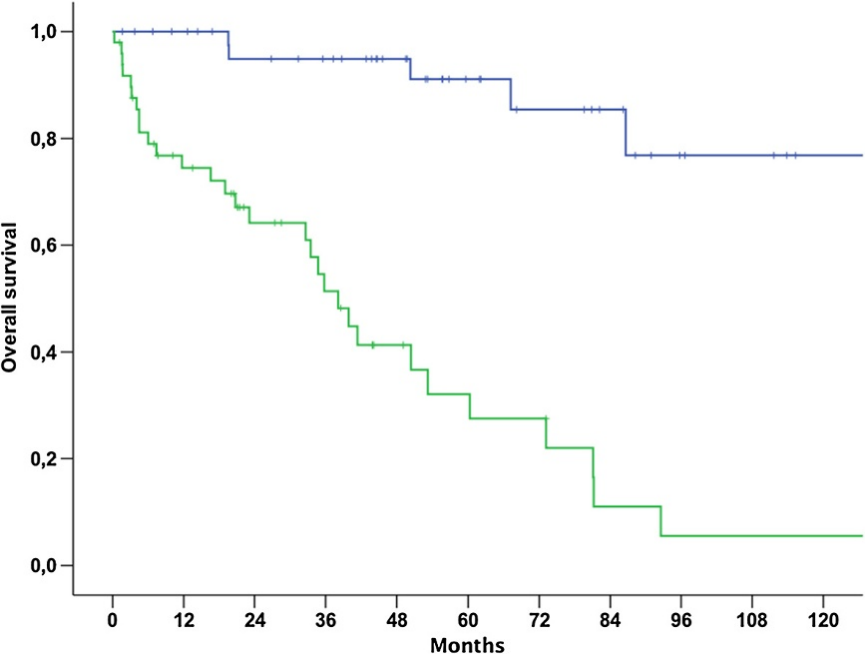
**Overall survival for patients with localized (blue line) vs. initially metastasized (green line) NULMS and ULMS (p < 0.001); 5 yr OSR 91.3% vs. 29.6%.**

**Figure 3:**
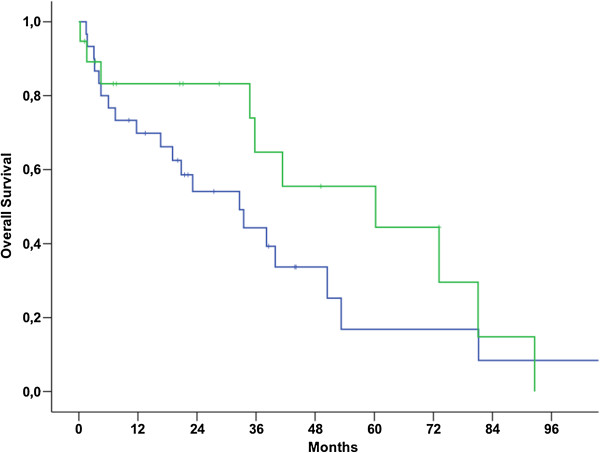
**Subgroup analysis: overall survival between initially metastatic NULMS (green curve) and ULMS (blue curve) (5-yr OS: NULMS: 44.4%; ULMS: 16.9%; p = 0.22).**

Median OS for patients with NULMS and ULMS was 92.6 (79.7-105.4 95% CI) and 50.4 (34.8-66.0 95% CI) months, respectively (p = 0.006) (Figure 1). Patients with initial metastatic disease showed unfavorable outcome when compared to patients with initial localized disease as shown in Table 2 and Figure 2 (p < 0.001). Therefore, these parameters were included into a multivariate model. In this model, initial metastatic disease remained an independent prognostic factor (p < 0.001, 95% CI: 3.8-26.5), whereas tumor location (NULMS vs ULMS) did not show any prognostic effect (p = 0.1, 95% CI: 0.3-1.1). A subgroup analysis between initial metastatic NULMS und ULMS showed no significant difference in OS, but a trend towards worse outcome for ULMS could be observed (NULMS: 5-yr OS rate: 44.4%; ULMS: 5-yr OS rate: 16.9%; p = 0.22) (Figure 3).

**Table 2:** **Univariate survival analyses with 5-year overall survival rates**

		5-yr OSR	p value
**Location**			0.006
	Non-uterine	82.6%	
	Uterine	41.2%	
**Gender**			0.07
	Male	86.2%	
	Female	53.4%	
**Size**			0.71
	≤5 cm	66.7%	
	5-10 cm	57.9%	
	>10 cm	32.4%	
**Initial metastatic**			<0.001
	No	91.3%	
	Yes	29.6%	
**Grading**			0.06
	G1 + 2	73.6%	
	G3	50.5%	
**Age**			0.06
	>56.6a	75.6%	
	≤56.6a	49.2%	
**Gender (NULMS)**			0.96
	Female	77.9%	
	Male	76.7%	

5-yr OSR = 5-year Overall survival rate; NULMS = non-uterine LMS.

**Figure 4:**
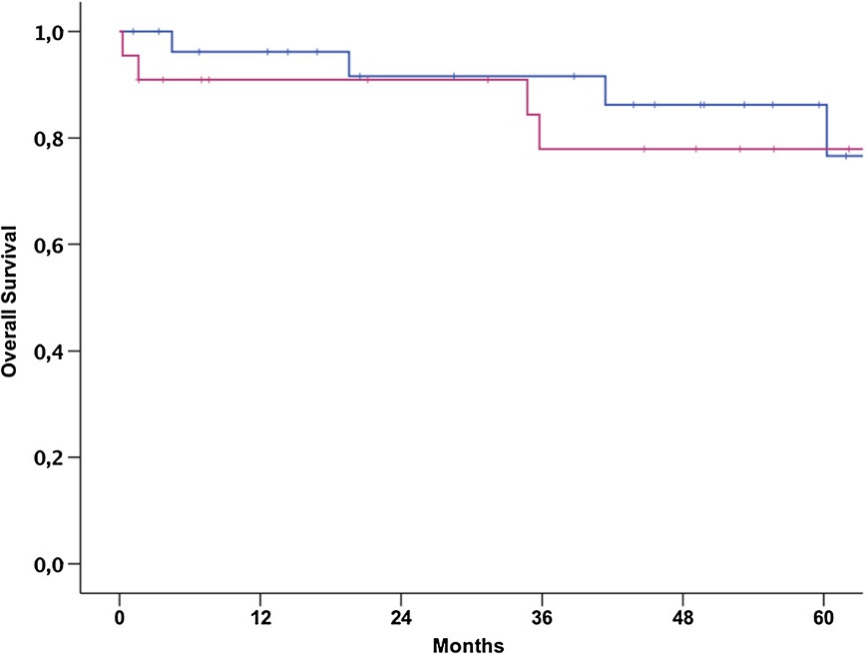
**Impact of gender on overall survival of patients with NULMS (p = 0.96); 5 yr OSR 76.6% (male) (blue curve) vs. 77.9% (female) (pink curve).**

**Figure 5:**
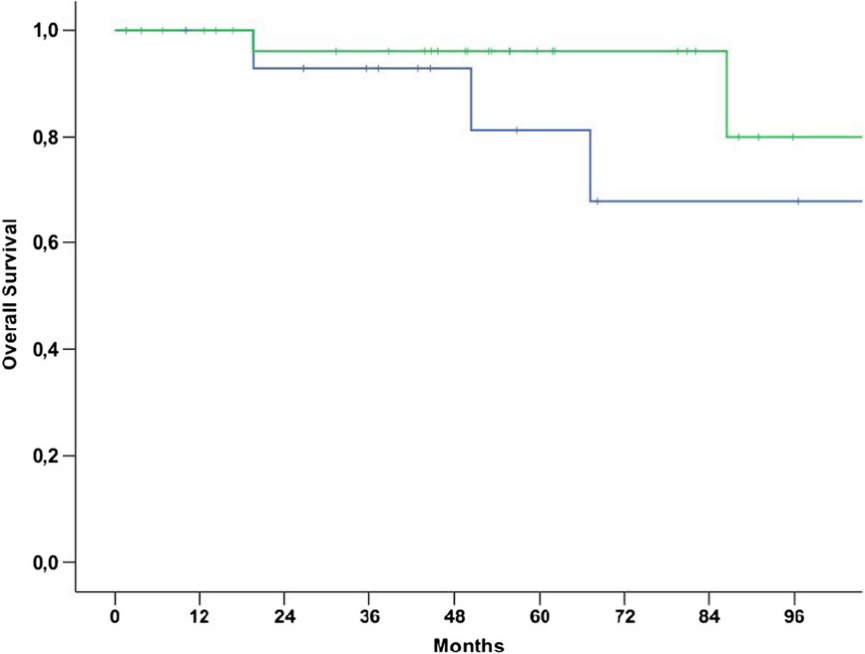
**Subgroup analysis: overall survival between initially localized NULMS (green curve) and ULMS (blue curve) (5-yr OS: NULMS: 96.0%; ULMS: 81.3%; p = 0.27).**

**Figure 6:**
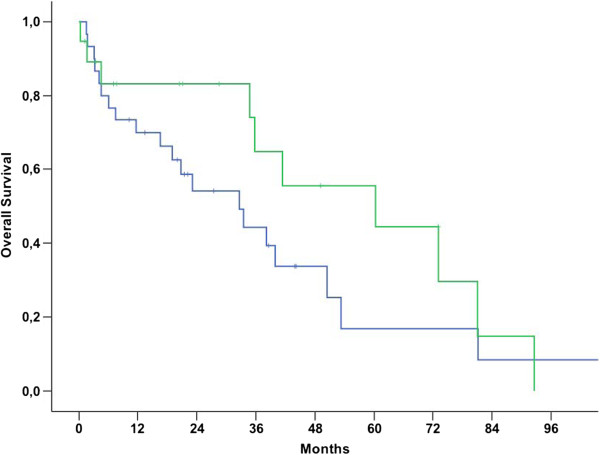
**Subgroup analysis: overall survival between initially metastatic NULMS (green curve) and ULMS (blue curve) (5-yr OS: NULMS: 44.4%; ULMS: 16.9%; p = 0.22).**

Gender, tumor size, grading and age were not shown to be prognostic parameters for overall survival, as outlined in Table 2. In order to investigate the effect of age on prognosis of patients with ULMS a subgroup was performed. Results showed that younger women had impaired prognosis, just as in the complete cohort of patients (p = 0.048). A subgroup analysis was performed in all patients with NULMS in order to investigate a potential prognostic effect of gender as previously described [16]. There was no survival-difference observed according to gender (p = 0.96) (Figures 4, 5 and 6).

## Discussion

In this retrospective analysis comparing the clinical outcome of patients with NULMS and ULMS, we found significant differences in terms of clinical-pathological parameters and OS between these two groups. Patients with ULMS were found to have larger tumors and initial metastatic disease was more frequently observed at time of diagnosis. In multivariate analysis, initial metastatic disease was associated with worse OS for patients with ULMS and therefore seems to be the main cause for the unfavorable prognosis of patients with ULMS.

At time of diagnosis patients with NULMS presented with smaller tumors when compared to ULMS. This is not surprising as NULMS located at the extremities typically cause early symptoms such as swelling. In contrast ULMS and abdominal LMS cause unspecific abdominal symptoms and are frequently incidental findings at time of hysterectomy or myomectomy [17]. More advanced disease was also reflected by a higher rate of metastatic disease in ULMS. Interestingly, the rate of metastatic disease was high in our study cohort. This finding might be related to the fact that our institution is a reference center for sarcoma patients and more patients presented at our department with advanced disease. Interestingly, patients in our study with ULMS were younger when compared to patients with NULMS. This might be attributable to differences in tumor biology.

Another reason for the worse outcome may be the different gene expression between uterine and other LMSs, as discussed previously [18]. Tumor tissue of ULMS expresses different genes than NULMS. These genes are ESR1, HOXA10, PBX1 and FAT, which are regulators for urogenital differentiation, development and growth [19]. Another recent published work investigated different gene expression in primary tumor tissue and metastases of ULMS. The authors concluded different gene expression between these two subgroups [20]. NULMS are characterized by different gene alterations, complex karyotypes with numerous gains and losses. Some of these aberrations are associated with poor prognosis, such as p53 gene mutation, p16 inactivation, RASSF1A increase risk of tumor related death [21].

Furthermore, NULMS patients had a longer OS when compared to ULMS patients (p = 0.006). One explanation might be that ULMS are diagnosed at more advanced stage, like in our study population. Oosten et al. investigated for the first time the difference according to OS in patients with un-resectable or metastatic NULMS and ULMS. No statistically significant differences in outcomes (p = 0.47) were found. Only 36% of all patients with ULMS presented with distant metastases, when compared to our patients (67%). Also Salas and colleagues described that patient’s age (≥55a), tumor grade (G3), wide surgical resection margins, and histological subtype (angiosarcoma) are factors influencing OS in different superficial STS patients [22]. In contrast, our report revealed that younger patients showed impaired outcome. This might partly be explained by the fact that women with ULMS were younger than patients with NULMS. Of note, this seems unreasonable as a subgroup analysis of the influence of age on OS in women with ULMS showed that younger women had impaired prognosis, just as in the complete cohort of patients. Another reason might be that tumors in younger patients show more aggressive behavior as described in other malignancies such as gastric cancer [23].

In this study, patients with ULMS showed impaired survival when compared to NULMS. As we found a higher rate of initial metastatic disease in ULMS and unfavorable outcome for patients with metastases in both univariate and multivariable survival analysis, uterine tumor location rather seems to be a confounding variable than an independent prognosticator. Nevertheless, in a subgroup analysis of patients with metastatic disease we observed a (non-significant) trend towards impaired survival in patients with ULMS. The authors are aware that the lack of statistical significance warrants careful interpretation and validation in larger cohorts.

Male patients showed improved OS in our study. Interestingly gender did not influence outcome in patients with NULMS (Figure 4). Therefore it seems likely that female gender is a confounder for uterine tumor location and that gender-specific aspects such as chemosensitivity play a minor role. Nevertheless, our findings are in contrast to the results reported in the study by Oosten et al., where male patients with NULMS showed impaired outcome when compared to female NULMS patients [16]. Based on their findings the authors had previously suggested stratifying future clinical trials according to gender. Only OS for female patients with NULMS seemed to be longer when compared to male patients and female patients with ULMS. It has to be mentioned that in this trial the outcome was investigated after first line chemotherapy [16].

Various chemotherapy regimens in the front line as well in the palliative setting showed promising effect in first line as well in advanced disease for patients with NULMS [5–7] as well as ULMS [10, 12]. In our study the similar adjuvant systemic treatment approaches were used for both patients with NULMS and ULMS. It has to be underlined that in our study no standardized chemotherapy protocol was used and that several lines of systemic therapy were administered based on physicians choice, as it is typical for data generated from retrospective analysis. Studies suggest that ULMS show differences with regard to chemotherapy sensitivity compared to other STS types [19]. Furthermore, in the present study OS in our patients with NULMS and ULMS was longer when compared to the study by Oosten at al. [16] A potential explanation for our findings might be, that patients treated at our institution frequently received several lines of treatment. Furthermore additional active drugs, such as trabectedin [7, 24] and pazopanib [25], are approved and available in selected European institutions. Another reason may be, that our patients received more treatment lines (up to 4 different cycles of chemotherapy) during their medical history.

There are some study limitations in our study. On the one hand, it was a retrospective analysis, thus response rates due to various chemotherapy lines are limited and cannot be compared with response rates of other prospective trials. On the other hand, our study population is heterogeneous which is different to prospective trials.

## Conclusions

NULMS and ULMS showed differences in OS in our study cohort. Patients with ULMS were found to have larger tumors and initial metastatic disease was more frequently observed at time of diagnosis. In multivariate analysis, initial metastatic disease was associated with worse OS for patients with ULMS and therefore seems to be the main cause for the unfavorable prognosis of patients with ULMS. Future studies investigating the differences between ULMS and NULMS are warranted in order to validate our findings. Due to the differences in clinical presentation and outcome it seems reasonable that ULMS should be distinguished from NULMS for future clinical trials.

## Abbreviations

LMS: Leiomyosarcoma
NULMS: Non-uterine leiomyosarcoma
ULMS: Uterine leiomyosarcoma
OS: Overall survival
FNCLCC: French Federation of Cancer Centers Sarcoma Group
FIGO: International federation of gynecology and obstetrics
CT: Computed tomography
MRI: Magnetic resonance imagining.
